# Method development and validation of potent pyrimidine derivative by UV-VIS spectrophotometer

**DOI:** 10.1186/s13588-014-0015-9

**Published:** 2014-12-05

**Authors:** Anshu Chaudhary, Anoop Singh, Prabhakar Kumar Verma

**Affiliations:** 1 NIMS University, Shobha Nagar, Jaipur, 303001 Rajasthan India; 2 Vishveshwarya Institute of Medical Science, Gautambudh Nagar, 203207 Uttar Pradesh India; 3 Department of Pharmaceutical Sciences, M.D. University, Rohtak, 124001 Haryana India

**Keywords:** Pyrimidine, Derivative, UV-VIS spectroscopy, Validation

## Abstract

**Background:**

A rapid and sensitive ultraviolet-visible (UV-VIS) spectroscopic method was developed for the estimation of pyrimidine derivative 6-Bromo-3-(6-(2,6-dichlorophenyl)-2-(morpolinomethylamino) pyrimidine4-yl) -2H-chromen-2-one (BT_10_M) in bulk form.

**Results:**

Pyrimidine derivative was monitored at 275 nm with UV detection, and there is no interference of diluents at 275 nm. The method was found to be linear in the range of 50 to 150 μg/ml. The accuracy and precision were determined and validated statistically. The method was validated as a guideline.

**Conclusions:**

The results showed that the proposed method is suitable for the accurate, precise, and rapid determination of pyrimidine derivative.

**Graphical Abstract:**
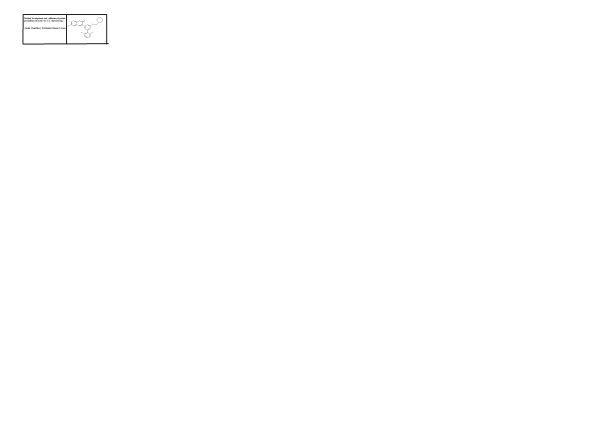
Method development and validation of potent pyrimidine derivative by UV spectroscopy.

**Electronic supplementary material:**

The online version of this article (doi:10.1186/s13588-014-0015-9) contains supplementary material, which is available to authorized users.

## Background

**Scheme 1:**
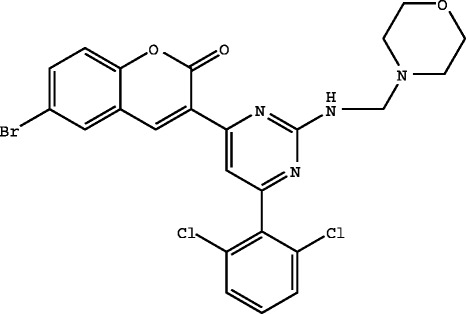
**Chemical structure of BT**_**10**_ **M.**

Nitrogen containing heterocyclic ring such as pyrimidine is a promising structural moiety for drug design. Pyrimidine derivatives form a component in various useful drugs and are associated with many biological and therapeutic activities. Condensed pyrimidines have been reported as antimicrobial [1]–[3], anti-inflammatory [4],[5], analgesic [6],[7], anticancer [8]–[10], anti-HIV [11], antitubercular, antimalarial, diuretic, and cardiovascular disease [12] (Scheme 1).

The present work is a synthesis, a biological evaluation and validation of novel pyrimidine derivatives. Research workers have synthesized 50 pyrimidine derivatives (T_1_M-T_10_M, T_1_P-T_10_P, BT_1_M-BT_10_M, BT_1_P-BT_10_P, CT_1_M-CT_5_M, and CT_1_P-CT_5_P). Among them, BT_10_M exhibited maximum antimicrobial, anti-inflammatory, and analgesic activity. Hence, a validation study was done on BT_10_M. BT_10_M is chemically [6-Bromo-3-(6-(2,6-dichlorophenyl)-2-(morpolinomethylamino) pyrimidine4-yl) -2H-chromen-2-one]. It is a yellow crystalline powder with a molecular formula of C_24_H_19_BrCl_2_N_4_O_3_ and a molecular weight of 562.24. It is a potent antimicrobial, analgesic and anti-inflammatory agent among all the synthesized derivatives. Hence, the aim of present investigation is to develop a simpler, rapid, and cost-effective analytical method for the determination of pyrimidine derivative (BT_10_M) in bulk dosage form suitable for routine quality control analysis.

Method validation is the process used to confirm that analytical procedure employed for a specific test is suitable for its intended use. It is an integral part of any good analytical practice. Methods need to be validated or revalidated [13].

## Methods

### Chemical and reagent

BT_10_M was synthesized by research workers and then validated. Methanol and acetonitrile (1:1) were used throughout spectrophotometric method development and validation.

### Instrumentation

The method was performed on a double-beam ultraviolet-visible (UV-VIS) spectrophotometer (Shimadzu model 1700 (Shimadzu, Kyoto, Japan)) having two matched quartz cells with a 1-cm light path.

### Determination of maximum wavelength (*λ*_max_), methodology, and sample preparation

About 50 mg of BT_10_M was weighed accurately and transferred into a 50-ml volumetric flask and dissolved in 25 ml of methanol and acetonitrile (1:1) and made up to the volume with the same solvent mixture to give a standard concentration of 1,000 μg/ml. Transfer 5 ml of above solution into the 50-ml volumetric flask, dilute, and made up to the volume with the same solvent mixture to get a standard concentration of 100 μg/ml. This solution was scanned against a blank over the entire UV-VIS wavelength of 200 to 400. Based on the spectrum, a *λ*_max_ of 275 nm was selected for further analysis.

## Results

The method was validated with respect to linearity, accuracy, precision, specificity, robustness, ruggedness, LOD, and LOQ in Table 1.

**Table 1:** **Validation summary**

Serial number	Parameters	Acceptance criteria	Observation
01	Precision		
	(a) System precision %RSD	NMT 1.5%	0.0968
	(b) Method precision %RSD	NMT 1.5%	0.27995
02	Specificity	No considerable absorbance of any other component of formulation at *λ*_max_ of analyte or at detection wavelength	No absorbance observed at 275 nm
03	Accuracy (by recovery)		
	% Recovery	100% ± 2%	100.12%
	%RSD	NMT 1.5%	1.1777%
	% Deviation from accuracy	±1.5%	080%: +01.46
			100%: −00.77
			120%: −00.32
04	Linearity	Coefficient of correlation	*r*^2^: 0.997
		(*r*^2^) NLT 0.998	
05	Ruggedness	%RSD: NMT 1.5%	%RSD: 0.1572
06	Robustness	%RSD: NMT 1.5%	Original condition: +0.72
			Changed condition: −0.82
07	Limit of detection and limit of quantitation	LOD	145.2 mg
		LOQ	440.00 mg

NMT, not more than; NLT, not less than.

## Discussion

**Figure 1:**
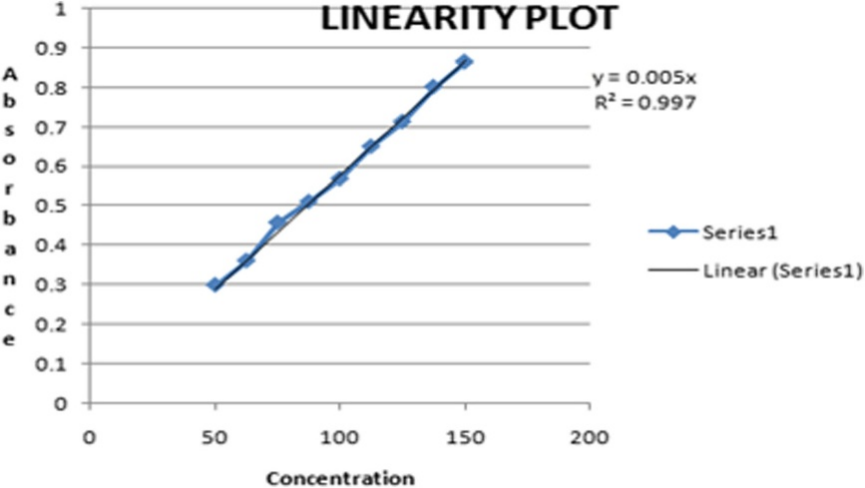
**Linearity plot.** The BT10M absorbance response in the concentration range of about 50 mcg/ml to 150 mcg/ml was found to be linear to the analyte concentration in the solution with a correlation coefficient (r2) of 0.997.

The method was validated with respect to linearity, accuracy, precision, specificity, robustness, ruggedness, LOD, and LOQ. The method was established according to the International Conference on Harmonisation of Technical Requirements for Registration of Pharmaceuticals for Human Use (ICH) guidelines. BT_10_M exhibited maximum absorption at 275 nm and obeyed Beer's law in the concentration range of 50 to 150 μg/ml. The proposed method for the determination of BT_10_M showed linear regression *y* = 0.005x + 0.025 with a coefficient correlation (*r*^2^) of 0.997 (Figure 1). The precision was determined by the relative standard deviation of the six-assay sample of BT_10_M, and the assay of each was calculated and the obtained relative standard deviation of % assay was less than 1.5%. The percentage recovery for BT_10_M was found in the range of 98.97% to 99.83% which indicates that the developed method was simple, rapid, and precise. LOD was found to be 145.2 and limit of quantitation to be 440.0. The proposed method will be suitable for the analysis of newly synthesized pyrimidine derivative (BT_10_M) in bulk dosage form.

## Experimental

### Validation

The methods were validated with respect to linearity, accuracy, precision, specificity, ruggedness, robustness, limit of detection (LOD), and limit of quantitation (LOQ).

#### Linearity

The linearity of an analytical method is its ability to elicit test results that are directly, or by a well-defined mathematical transformation, proportional to the concentration of analyte in the samples within a given range. For assay determination, the concentration of BT_10_M is 100 μg/ml. So the working range of analyte was set between 50, 62.5, 75, 87.5, 100, 112.5, 125, 137.5 and 150 μg/ml to show the linearity of the curve obtained. The observations and calibration curve are shown in Table 2 and Figure 1.

**Table 2:** **Linearity table of BT**_**10**_ **M in working standard**

Serial number	Approximate concentration (μg/ml)	Average absorbance at 275 nm
1	50.0	0.299
2	62.5	0.361
3	75.0	0.458
4	87.5	0.511
5	100.0	0.569
6	112.5	0.651
7	125.0	0.714
8	137.5	0.803
9	150.0	0.866

#### Accuracy (by recovery test)

Accuracy of method is by shown by recovery study and spiking working standard in the placebo at levels 80%, 100%, and 120% of the working standard. Recovery study was performed by spiking in BT_10_M to the placebo at levels 80%, 100%, and 120% of working standard. The samples were prepared according to the assay procedure. The results are shown in Tables 3 and 4.

**Table 3:** **Accuracy reading**

Level (Approximate)	Standard added (mg)	Absorbance at 275 nm	Standard recovered (mg)	%Recovery	Mean recovery	%RSD
80%	40.5	0.458	40.43	99.83	99.37%	0.4366
		0.451				
		0.459				
100%	50.8	0.567	50.28	098.97		
		0.561				
		0.574				
120%	60.1	0.670	59.68	099.30		
		0.676				
		0.673

**Table 4:** **Deviation from recovery**

Level (Approximate)	Actual concentration (μg/ml)	Concentration calculated (mg/ml)	Accuracy (%)	% Deviation
80%	080.75	081.9289	101.46	+01.46
100%	100.40	099.6269	099.23	−00.77
120%	121.05	120.6626	099.68	−00.32

The percentage recovery for BT_10_M was found in the range of 98.97% to 99.83% with an overall relative standard deviation (%RSD) of 0.4366. From the data obtained which was given in Table 3, the method was found to be accurate. Formula of standard deviation was SD = (xi − *x*/*n* − 1)1/2 if *n* is very large. In case of very small data, SD = (xi − *x*/*n*)1/2.

#### Precision

The precision of an analytical method is the degree of agreement among individual test results when the method is applied repeatedly to multiple sampling of homogenous sample. The precision of an analytical method is usually expressed as the standard deviation or relative standard of a series of measurements. Assay preparation and standard preparation were prepared as per method of analysis of six BT_10_M assay sample preparations as per the experimental conditions in method of analysis. Calculated percent of BT_10_M in each assay sample percent by spectrophotometry and the results and observation are summarized in Tables 5 and 6.

**Table 5:** **System precision data of BT**_**10**_ **M working standard solution**

Serial number	Absorbance at 275 nm
1	0.566
2	0.565
3	0.565
4	0.566
5	0.566
6	0.565
Average	0.565
%RSD	0.0968

Weight of BT_10_M WS = 50.0 mg.

**Table 6:** **Method precision data for estimation of BT**_**10**_ **M**

Assay	Sample weight (mg)	Absorbance	% Assay
1	50.2	0.576	101.75
2	50.4	0.576	101.29
3	50.2	0.572	101.02
4	50.8	0.580	101.22
5	50.0	0.569	100.96
6	50.7	0.579	101.37
Average			101.2683
%RSD			0.27995

#### Assay

$$\%\;\mathrm{Assay}=\frac{\mathrm{Abs}.\;\mathrm{of}\;Smp.}{\mathrm{Abs}.\;\mathrm{of}\;\mathrm{standard}}\times \frac{Wt.\;\mathrm{of}\;\mathrm{standard}}{Dil.\;\mathrm{factor}}\times \frac{Dil.\;\mathrm{factor}}{Wt.\;\mathrm{of}\;Smp}\times 100$$

The precision will be determined by the relative standard deviation of the six-assay sample of BT_10_M, and the assay of each is calculated and the obtained relative standard deviation of % assay should be less than 1.5%. The %RSD shows that precision of the method was satisfactory.

#### Specificity

**Figure 2:**
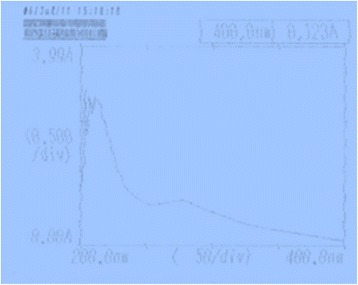
**Spectrum of BT**_**10**_ **M.**

Specificity study is designed to prove that the BT_10_M in the solution gives maximum absorbance at wavelength 275 nm, and there is no interference from the solvent. The purpose of this study is to establish the fact that inherent chemical stability of the molecule remains intact during its existence. If any degradation product formed, it can be monitored and resolved to quantify the nature and extent of degradation. For this, the spectrum of BT_10_M, placebos are studied. The sample preparation is as per methodology. The spectrum of BT_10_M is shown in Figure 2.

For the spectrophotometric method, no other component of formulation shows considerable absorbance at the *λ*_max_ of the analyte or at the detection wavelength of the subject analyte. In this case of BT_10_M, the detection wavelength is 275 nm. The placebo solution under the same condition does not show any absorbance at 275 nm.

#### Ruggedness

The ruggedness of the analytical method is the degree of reproducibility of test results obtained by the analysis of the sample on different days, by different chemist using different instruments. In this study, two individual assay sample preparations of BT_10_M drug product were prepared by different chemists for analysis. Six (6) replicate observations of the same standard solution were obtained as well as six observations of different sample solution were recorded. The assay percentage of each sample was calculated in each case. The results are summarized in Tables 5, 6, 7, 8, 9.

**Table 7:** **System precision data of BT**_**10**_ **M working standard solution**

Serial number	Absorbance at 275 nm
1	0.559
2	0.561
3	0.560
4	0.561
5	0.560
6	0.561
Average	0.560
%RSD	0.14571

Weight of BT_10_M: 49.8 mg.

**Table 8:** **Method precision data for BT**_**10**_ **M**

Assay	Sample weight (mg)	Absorbance	% Assay
1	50.2	0.566	100.27
2	50.5	0.572	100.73
3	50.8	0.582	101.94
4	50.7	0.578	101.45
5	50.2	0.568	100.64
6	50.9	0.579	101.23
Average			101.0433
%RSD			0.6041

**Table 9:** **Mean % assay and %RSD**

Chemist A	Chemist B	Average	%RSD
101.2683	101.0433	101.1558	0.1572

The ruggedness (inter-day precision) will be determined by the relative standard deviation of the results of assay of two different chemists on different days.

#### Robustness

The robustness of an analytical method is a measure of its capacity to remain unaffected by small but deliberate variations in method parameters and provides an indication of its reliability under normal usage. Method robustness was determined by analyzing the same sample at normal operating conditions and also by changing some operating analytical conditions. The result and observation are summarized in Table 10.

**Table 10:** **Percentage deviation for sample under both conditions**

Parameter	Original condition	Changed conditions
Dilution medium	Acetonitrile:methanol	Acetonirile:methanol
	1:1	1:2
Assay in %	101.98	100.44
% Deviation from mean assay value obtained in precision studies	+00.72	−00.82

The robustness will be determined by the relative standard deviation of the results of assay of two different conditions by a change in original parameter.

#### Limit of detection and limit of quantitation

##### Limit of detection

LOD is defined as the lowest concentration of an analyte in a sample that can be detected, not quantified.

##### Limit of quantitation

The lowest concentration of an analyte in a sample that can be determined with acceptable precision and accuracy under the stated operational conditions of the method.

$$\begin{array}{c} LOD:\;3.3\left(SD\right)/\mathrm{Slope}\\ 3.3\left(0.200\right)/0.005\;\left[SD=0.2200\right]\\ =145.2\;\mathrm{mg}\\ 2.\;LOQ:\;10\left(SD\right)/\mathrm{Slope}\\ 10\left(0.2200\right)/0.005\\ =440.00\;\mathrm{mg} \end{array}$$

## Conclusions

All the validation parameters for all the developed methods were studied as per ICH guidelines. All the methods were found to be accurate, simple, specific, selective, precise, and reproducible. Hence, the method can be used for routine analysis of BT_10_M in bulk dosage form.

## Authors’ original submitted files for images

Below are the links to the authors’ original submitted files for images. Authors’ original file for figure 1 Authors’ original file for figure 2 Authors’ original file for figure 3
