# Supplementary figures and images for: Method development and validation of potent pyrimidine derivative by UV-VIS spectrophotometer
Source: Org Med Chem Lett. 2014 Dec 5;4:15. doi: 10.1186/s13588-014-0015-9 (PMC4970434; doi:10.1186/s13588-014-0015-9)

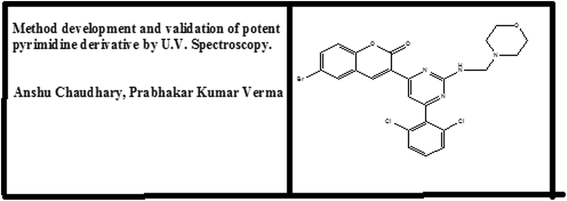

Supplement: Supplementary file 1 — Authors’ original file for figure 1 [file 13588_2014_15_MOESM1_ESM.gif]

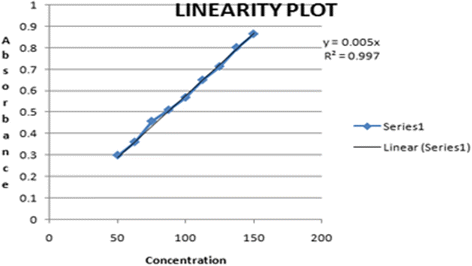

Supplement: Supplementary file 2 — Authors’ original file for figure 2 [file 13588_2014_15_MOESM2_ESM.gif]

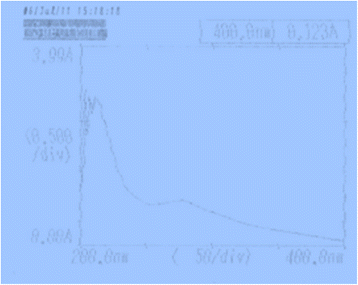

Supplement: Supplementary file 3 — Authors’ original file for figure 3 [file 13588_2014_15_MOESM3_ESM.gif]
